# Development and validation of a nomogram to predict the recurrence of hepatocellular carcinoma patients with dynamic changes in AFP undergoing locoregional treatments

**DOI:** 10.3389/fonc.2023.1206345

**Published:** 2023-08-28

**Authors:** Yu Sun, Yiqi Xiong, Qi Wang, Wenying Qiao, Honghai Zhang, Yonghong Zhang

**Affiliations:** ^1^ Interventional Therapy Center for Oncology, Beijing You’an Hospital, Capital Medical University, Beijing, China; ^2^ Research Center for Biomedical Resources, Beijing You’an Hospital, Capital Medical University, Beijing, China

**Keywords:** Hepatocellular carcinoma, alpha-fetoprotein (AFP), TACE, ablation, nomogram, recurrence

## Abstract

**Background:**

Serum alpha-fetoprotein (AFP) is an important clinical indicator for screening, diagnosis, and prognosis of primary hepatocellular carcinoma (HCC). Our team’s previous study showed that patients with negative AFP at baseline and positive AFP at relapse had a worse prognosis (N-P). Therefore, the aim of our study was to develop and validate a nomogram for this group of patients.

**Methods:**

A total of 513 patients with HCC who received locoregional treatments at Beijing You’an Hospital, Capital Medical University, from January 2012 to December 2019 were prospectively enrolled. Patients admitted from 2012 to 2015 were assigned to the training cohort (n = 335), while 2016 to 2019 were in the validation cohort (n =183). The clinical and pathological features of patients were collected, and independent risk factors were identified using univariate and multivariate Cox regression analysis as a basis for developing a nomogram. The performance of the nomogram was evaluated by C-index, receiver operating characteristic (ROC) curves, calibration curves, and decision curve analysis (DCA) curves in the training and validation cohorts.

**Results:**

The content of the nomogram includes gender, tumor number, tumor size, lymphocyte, direct bilirubin (DBIL), gamma-glutamyl transferase (GGT), and prealbumin. The C-index (0.717 and 0.752) and 1-, 3-, and 5-year AUCs (0.721, 0.825, 0.845, and 0.740, 0.868, 0.837) of the training and validation cohorts proved the good predictive performance of the nomogram. Calibration curves and DCA curves suggested accuracy and net clinical benefit rates. The nomogram enabled to classify of patients with dynamic changes in AFP into three groups according to the risk of recurrence: low risk, intermediate risk, and high risk. There was a statistically significant difference in RFS between the three groups in the training and validation cohorts (P<0.001).

**Conclusion:**

The nomogram developed and validated in this study had good predictive power for patients with dynamic changes in AFP.

## 1 Introduction

In 2020, almost 906000 people worldwide were diagnosed with liver cancer, with the most common category being hepatocellular carcinoma (HCC) which is the third leading cause of cancer death in the world (1–3). China has the highest incidence rate, accounting for more than half of new cases and deaths from liver cancer worldwide (4, 5). Only less than 40% of patients in China were diagnosed with early-stage liver cancer (6). Surgery, liver transplantation, and local therapy are recommended for patients with early-stage liver cancer. Previous studies have shown that ablation therapy has similar survival rates but fewer complications compared with surgery in small liver cancer (7). However, the recurrence rate after ablation therapy is high, with a five-year recurrence rate of 50%-70% (8–10). Transcatheter arterial chemoembolization (TACE) is the only guideline-recommended global standard of care for intermediate-stage HCC, while the median progression-free survival (mPFS) is only five months for intermediate-stage liver cancer (8, 10). Therefore, the prognosis and treatment of liver cancer are fundamental important public health issues in China.

Since its identification in the 1960s, alpha-fetoprotein(AFP), which was first described as a marker for HCC, has become the most widely used biomarker for the diagnosis and prognosis of HCC (11). But AFP has a high specificity (80%-90%) and a low sensitivity (40%-60%) (12), and these patients with normal AFP (<20ng/ml) in the baseline are called AFP-negative HCC patients (AFP-NHCC). As an essential diagnosis criterion, AFP-negativity can affect the early diagnosis and treatment of patients, thus affecting the prognosis of patients (13, 14). Our preliminary research showed that among AFP-NHCC patients, those who turned positive at relapse had a 130% increased risk of death compared with those who remained negative AFP at recurrence (15). Because of the special clinicopathological features and prognosis of patients with dynamic changes in AFP (N-P, negative at baseline and positive at relapse) (16), reliable prediction models are needed to guide the clinical treatment of these patients.

At present, it has been proved that some biomarkers, such as ALBI, MLR, GGT, and GLR, are associated with the prognosis of patients with AFP-NHCC (17–20), and there are also predictive models of patients with AFP-NHCC models for surgical treatment (21–24). Yet, the predictive nomogram of recurrence for patients with dynamic changes in AFP who underwent ablation has been still lacking. Consequently, the purpose of our study is to develop and validate a reliable nomogram for this group of patients to more accurately guide the clinical decision.

## 2 Materials and methods

### 2.1 Patients

This study retrospectively evaluated 518 HCC patients aged 18-75 years old who underwent TACE combined with ablation at Beijing Youan Hospital from January 2012 to December 2019. The diagnosis of HCC was based on the guideline of the America Association for the Study of Liver Diseases (ASSLD) (1, 25). Patients admitted from January 1, 2012 to December 31, 2015 were assigned to the training cohort (n = 335), while January 1, 2016 to December 31, 2019 were in the validation cohort (n =183).

The inclusion criteria of patients were as follows: (1) early-stage HCC patient accepted TACE combined ablation achieved complete response. (2) Child-Pugh classification was class A or B. (3) AFP negative at baseline and positive at relapse (N-P). (4) All patients had not received any other therapeutics prior to ablation. The exclusion criteria included: (1) received other treatment before ablation; (2) advanced HCC; (3) with other primary malignancies; (4) clinical follow-up data incomplete.

Our study was approved by the Ethics Committee of Beijing Youan Hospital, Capital Medical University, and was conducted in accordance with the standards of the Declaration of Helsinki. The Ethics Committee considered that study as low-risk, so the requirement for informed consent of the patients was waived.

### 2.2 Clinicopathologic characteristics

Clinicopathologic Characteristics were collected from patients before surgery, including age, gender, etiology, tumor number, tumor size, Child-Pugh class, alanine aminotransferase (ALT), aspartate transaminase (AST), albumin (ALB), gamma-glutamyl transferase (GGT), total bilirubin (TBIL); direct bilirubin (DBIL), prealbumin, neutrophils (Neu), lymphocytes (Lym), and monocytes (Mon).

### 2.3 Treatment received

#### 2.3.1 TACE procedure

All TACE treatments were performed by two interventional radiologists. Under local anesthesia, the right femoral artery was punctured using a modified Seldinger technique. An arteriogram was carried out through a 5-F catheter (Terumo, Tokyo, Japan) to confirm portal vein patency and detect arterial supply to tumors. When applicable, a microcatheter was inserted into the blood-supply artery of the carcinoma to inject a mixture of doxorubicin (Pfizer Inc., New York, NY, USA) and lipiodol (Guerbet, Villepinte, France), followed by embolization using embolic materials, such as gelfoam or polyvinyl alcohol particles. The blood flow was monitored until complete vessel occlusion and flow wholly ceased. If the lesion is not completely necrotic and the active portion exceeds 50% of the baseline value, repeat embolization is required.

#### 2.3.2 Ablation procedure

Local ablation by qualified hepatologist was carried out within two weeks after TACE, guided by triple-phase computed tomography (CT) and magnetic resonance imaging (MRI). The size of the tumor decided the number of electrodes. Routine disinfection and local anesthesia around the puncture point were combined with intravenous analgesia and monitored anesthesia care. In the process of RFA, after measuring the baseline impedance, the power gradually increased from 80w to 200w to reach the maximum impedance. Cold brine was injected into the electrode chamber using a pump so that the tip temperature was consistently below 20°C. To achieve complete ablation, the range of ablation extended 0.5-1.0 cm to determine complete coverage; otherwise, the procedure was defined as an incomplete ablation. In order to prevent postoperative bleeding and tumor implantation along the needle track, the electrode was heated to 90°C-100°C and then pulled out. All patients underwent contrast-enhanced CT immediately after ablation to evaluate the technical success of the procedure and the development of any possible complications.

### 2.4 Follow-up

All patients were regularly follow-up in the section for outpatients. Responses were evaluated using CT and MRI at approximately 4-6 weeks post-treatment. In the first year following ablation, patients were examined every three months and then every six months thereafter. The follow-up involved blood tests, liver function, and imaging tests to detect tumor recurrence. Among the primary endpoints of this study was the recurrence-free survival (RFS), which was defined from the date of curative treatment to the first HCC recurrence or death from any cause.

### 2.5 Statistical analysis

Comparative analysis was done by t-test, chi-square test, and Mann-Whitney U test, with the purpose of providing median or counts and percentages to summarize baseline variables. Survival curves were drawn using the Kaplan-Meier method and compared using log-rank tests. Univariate and multivariate Cox regression analyses were performed to identify the independent risk factors for recurrence in AFP-NHCC patients undergoing ablation.

Following the above analyses, a nomogram was developed based on the Cox regression to predict recurrence. The validation cohort of the prognostic nomogram was verified. According to the nomogram scores, the patients were classified as low-risk, medium-risk, and high-risk groups, and their recurrence rates were predicted. The receiver operating characteristic (ROC) curve was drawn over time, and the area under the ROC curve (AUC) was calculated to the prognosis value of candidate factors. Calibration curves and the Hosmer-Lemeshow test were conducted to assess the predictive ability of the nomogram. Besides, decision curve analysis (DCA) was implemented to demonstrate the clinical utilization by quantifying the net benefits of the nomogram model in both training and validation cohorts.

All data were analyzed with SPSS (version 26.0, IBM, Armonk, NY, USA) and R software (version 4.1.2) in this study. Two-tailed tests were performed, and statistical significance was determined by P value of less than 0.05 (P<0.05).

## 3 Results

### 3.1 Patients characteristics

A total of 518 patients (**Figure S1**) with dynamic changes in AFP were screened between January 1, 2012 to December 31, 2019, divided by time into a training cohort (n=335) and a validation cohort (n=183). Up to the last follow-up date of January 1, 2022, the median follow-up time was 4.48 years (25~75th percentiles, 3.13~6.33 years).

There were no statistical differences in all baseline characteristics between the training cohort and the validation cohort (**Table 1**). Both in the training cohort and validation cohort, the majority of the patients were male (83.9% vs. 77.6%), and the average age was over 50 years (56.73 ± 8.85 vs. 56.95 ± 8.6). The HBV infection was the primary cause of HCC (91.9% vs. 89.6). Most patients were Child-Pugh A (74% vs. 79.2%), suggesting that the patients had good liver function. Regarding tumor characteristics, most tumors were solitary (67.2% vs. 70.5%) and tumor size was less than 3cm (62.7% vs. 66.7%, P=0.367). Radiofrequency ablation therapy (51.6% vs. 72.1%) was the primary ablation modality used for patients.

**Table 1:** Demographics and clinical characteristics for training and validation sets.

Characteristic	Primary cohort(N=335)	Validation cohort(N=183)	P value
Age	56.74 ± 8.85	56.95 ± 8.60	0.797
Gender Male Female	281(83.9%) 54(16.1%)	142(77.6%) 41(22.4%)	0.077
Hypertension Yes No	255(76.1%) 80(23.9%)	132(72.1%) 51(27.9%)	0.318
Diabetes Yes No	273(81.5%) 62(18.5%)	144(78.7.5) 39(21.3%)	0.479
Antiviral Yes No	157(46.9%) 178(53.1%)	88(48%) 95(52%)	0.581
Smoking Yes No	183(54.6%) 152(45.4%)	95(51.9%) 88(48.1%)	0.554
Drinking Yes No	207(61.8%) 128(38.2%)	120(65.6%) 63(34.4%)	0.394
ALD Yes No	271(80.9%) 64(19.1%)	137(74.9%) 46(25.1%)	0.109
Cirrhosis Yes No	62(18.5%) 273(81.5%)	35(19.1%) 148(80.9%)	0.712
Etiology HBV Alcohol	308(91.9%) 27(8.1%)	164(89.6%) 19(10.4%)	0.435
Child-Pugh class A B	248(74%) 87(26%)	145(79.2%) 38(20.8%)	0.186
BCLC stage 0 A B	105(31.3%) 189(56.4%) 41(12.2%)	66(36.1%) 103(54.3%) 14(7.6%)	0.207
T.N. Single Multiple	225(67.2%) 110(32.8%)	129(70.5%) 54(29.5%)	0.436
T.S. <3cm ≥3cm	210(62.7%) 125(37.3%)	122(66.7%) 61(33.3%)	0.367
Ablative modality RFA MWA AHC	173(51.6%) 82(24.5%) 80(23.9%)	132(72.1%) 46(25.1%) 5(2.8%)	0.061
WBC (10^9/L)	5.07 ± 2.22	5.32 ± 2.33	0.235
Neu (10^9/L)	3.21 ± 1.83	3.44 ± 1.96	0.189
Lym (10^9/L)	1.28 ± 0.67	1.32 ± 0.69	0.535
Mon (10^9/L)	0.43 ± 0.27	0.41 ± 0.23	0.575
Hb (g/L)	130.2 ± 19.14	130.44 ± 18.07	0.333
PLT (10^9/L)	122.51 ± 63.90	127.21 ± 67.21	0.432
ALT (U/L)	31.36 ± 21.85	28.83 ± 15.92	0.173
AST (U/L)	31.52 ± 15.37	29.9 ± 14.91	0.248
TBIL (umol/L)	18.85 ± 9.71	19.31 ± 9.68	0.612
DBIL (umol/L)	5.44 ± 3.96	5.88 ± 4.64	0.481
Total.alb (g/L)	64.90 ± 8.05	65.49 ± 7.67	0.419
Alb (g/L)	37.66 ± 4.99	37.45 ± 4.22	0.629
Globulin (g/L)	27.77 ± 5.01	28.27 ± 5.27	0.286
GGT (U/L)	64.61 ± 49.20	66.63 ± 58.29	0.677
ALP (U/L)	86.28 ± 34.03	85.25 ± 32.41	0.739
Palb (U/L)	125.33 ± 53.26	129.61 ± 39.66	0.795
INR	1.07 ± 0.12	1.14 ± 0.13	0.821
Fib (g/L)	2.77 ± 0.91	2.95 ± 1.05	0.145
PT (g/L)	12.25 ± 1.47	12.83 ± 1.47	0.231

ALD, alcoholic liver cancer; HBV, hepatitis B virus; BCLC, Barcelona Clinic Liver Cancer; T.N, tumor number; T.S, tumor size; RFA, radiofrequency ablation; MWA, microwave ablation; WBC, leukocyte; Neu, neutrophil; Lym, lymphocyte; Mon, monocyte; Hb, hemoglobin; PLT, platelet; ALT, alanine aminotransferase; AST, aspartate aminotransferase; TBIL, total bilirubin; DBIL, direct bilirubin; ALB, albumin; GGT, gamma glutamyl transferase; ALP, alkaline phosphatase; Palb, prealbumin; INR, international normalized radio; Fib, fibrous protein; PT, prothrombin time.

### 3.2 Independent prognostic factors of RFS

Univariate and multifactorial Cox regression analyses were performed to assess the association between clinical characteristics and RFS. Univariate Cox regression analysis showed that age, gender, Child-Pugh, BCLC stage, tumor size, tumor number, lymphocyte count, TBIL, DBIL, GGT, ALP, albumin, INR, and PT were associated with RFS. Multi-factors Cox regression analysis revealed that gender (HR: 2.52, 95% CI: 1.25-3.61), tumor size (HR: 1.62, 95%CI: 1.01-2.22), tumor number (HR: 1.21, 95%CI: 1.06-1.93), lymphocyte (HR: 1.21, 95% CI: 1.09-2.19), DBIL (HR: 1.05, 95%CI: 1.01-1.08), GGT (HR: 1.23, 95%CI: 1.01-1.46), and prealbumin (HR: 0.89, 95%CI: 0.61-0.97) were independent predictors for RFS (**Table 2**).

**Table 2:** Univariate and multivariate cox hazards analysis of the primary cohort.

Characteristic	Univariate analysis		Multivariate analysis	
	HR (95% CI)	P value	HR (95% CI)	P value
Age	0.99 (1-1.03)	**0.036**		
Gender	2.87 (1.23-4.53)	**<0.001**	2.52 (1.25-3.61)	**<0.001**
Hypertension	1.23 (0.61-1.09)	0.175		
Diabetes	1.13 (0.64-1.23)	0.471		
Antiviral	1.16 (0.67-1.1)	0.232		
Smoking	0.83 (0.94-1.54)	0.143		
Drinking	0.83 (0.94-1.55)	0.146		
ALD	0.94 (0.78-1.45)	0.706		
Cirrhosis	0.81 (0.89-1.71)	0.214		
Etiology	1.04 (0.85-1.09)	0.51		
Child-Pugh class	0.72 (1.05-1.82)	**0.019**		
BCLC stage	0.47 (1.7-2.62)	**<0.001**		
T.N.	1.47 (1.04-2.73)	**<0.001**	1.62 (1.01-2.22)	**0.004**
T.S.	1.53 (1.08-2.44)	**<0.001**	1.69 (1.06-1.93)	**0.017**
Ablative modality	1.08 (0.8-1.07)	0.298		
WBC (10^9/L)	1.02 (0.93-1.04)	0.494		
Neu (10^9/L)	1.0 (0.94-1.07)	0.914		
Lym (10^9/L)	1.26 (1.06-1.95)	**0.014**	1.21 (1.09-2.19)	**0.049**
Mon (10^9/L)	0.8 (0.81-1.95)	0.315		
Hb (g/L)	1.01 (0.99-1)	0.069		
PLT (10^9/L)	1.0 (0.98-1.2)	0.119		
ALT (U/L)	1.0 (0.98-1.1)	0.442		
AST (U/L)	0.99 (0.9-1.01)	0.097		
TBIL	0.98 (1.01-1.03)	**0.016**		
DBIL	1.06 (1.02-1.08)	**0.005**	1.05 ( 1.01-1.08)	**0.014**
Total.alb	1.01 (0.98-1.01)	0.201		
Alb (g/L)	1.05 (0.93-0.98)	**0.001**		
Glob (g/L)	0.98 (0.99-1.05)	0.072		
GGT(U/L)	1.12 (1.01-1.46)	**<0.001**	1.23 (1.00-1.31)	**0.003**
ALP (U/L)	1.0 (1.01-1.02)	**0.004**		
Palb (U/L)	0.79(0.69-0.98)	**<0.001**	0.89 (0.61-0.97)	**0.021**
INR	0.18 (2-15.37)	**0.001**		
Fib (g/L)	0.97 (0.9-1.19)	0.65		
PT (g/L)	0.87 (1.06-1.25)	**0.001**		

ALD, alcoholic liver cancer; HBV, hepatitis B virus; BCLC, Barcelona Clinic Liver Cancer; T.N, tumor number; T.S, tumor size; RFA, radiofrequency ablation; MWA, microwave ablation; WBC, leukocyte; Neu, neutrophil; Lym, lymphocyte; Mon, monocyte; Hb, hemoglobin; PLT, platelet; ALT, alanine aminotransferase; AST, aspartate aminotransferase; TBIL, total bilirubin; DBIL, direct bilirubin; ALB, albumin; Palb, prealbumin; GGT, gamma glutamyl transferase; ALP, alkaline phosphatase; INR, international normalized radio; Fib, fibrous protein; PT, prothrombin time.

Bold values represent statistically significant differences.

### 3.3 Develop the nomogram

**Figure 1:**
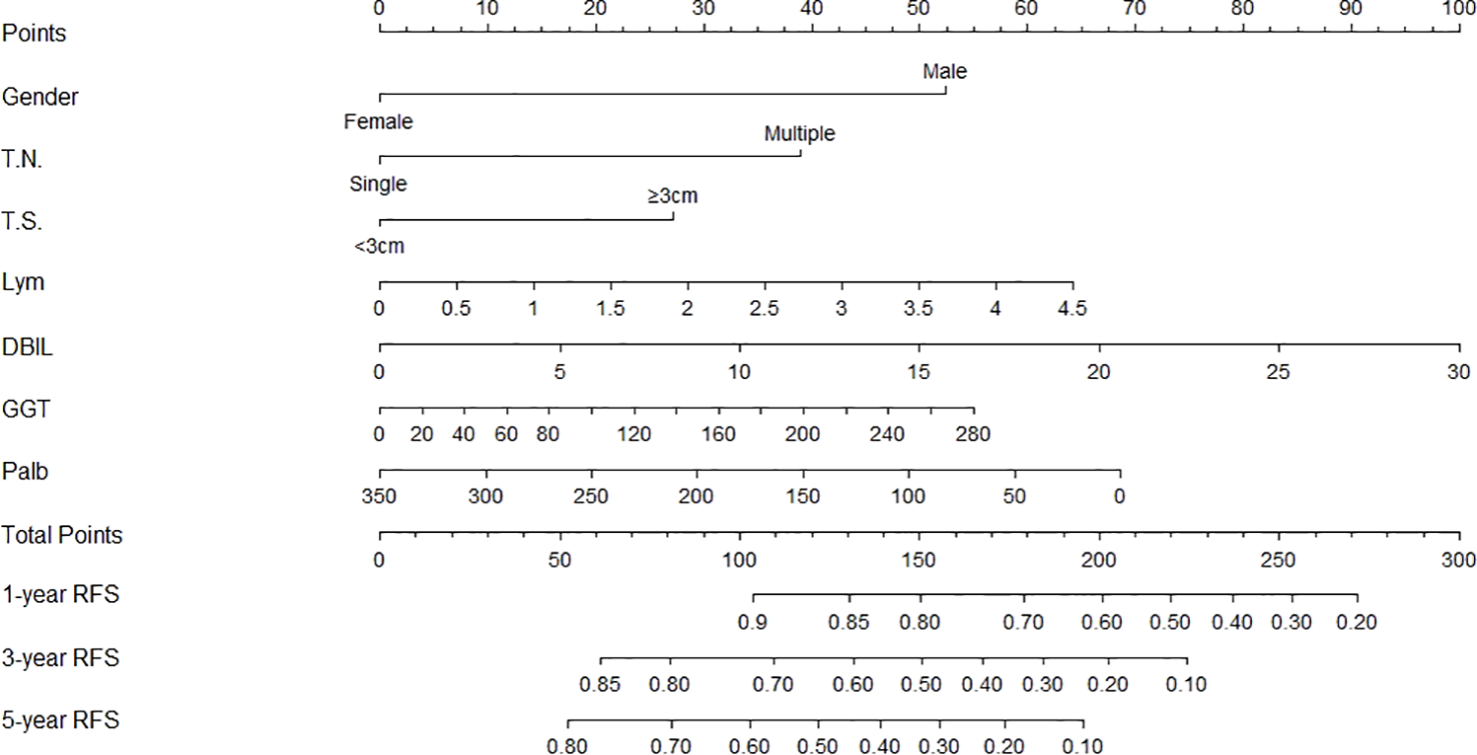
Nomogram, including gender, tumor number, tumor size, lymphocyte, DBIL, GGT, and prealbumin for one, three, and five years recurrence free survival (RFS) in HCC patients with dynamic changes in AFP. The nomogram is valued to obtain the probability of one, three, and five years recurrence by adding up the points identified on the points scale for each varible. T.N, tumor number; T.S, tumor size; DBIL, direct bilirubin; GGT, gamma glutamyl transferase; Palb, prealbumin.

**Figure 2:**
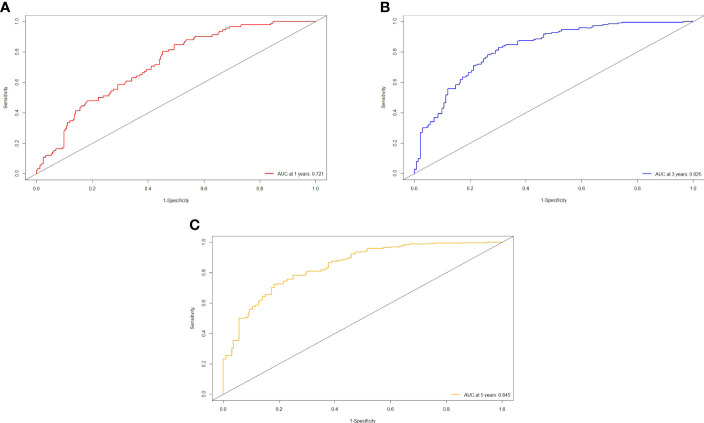
ROC curve of the nomogram in the training cohort. **(A)** The AUC for 1-year RFS was 0.721 in the training cohort. **(B)** The AUC for 3-year RFS was 0.825 in the training cohort. **(C)** The AUC for 5-year was 0.845 in the training cohort.

**Figure 3:**
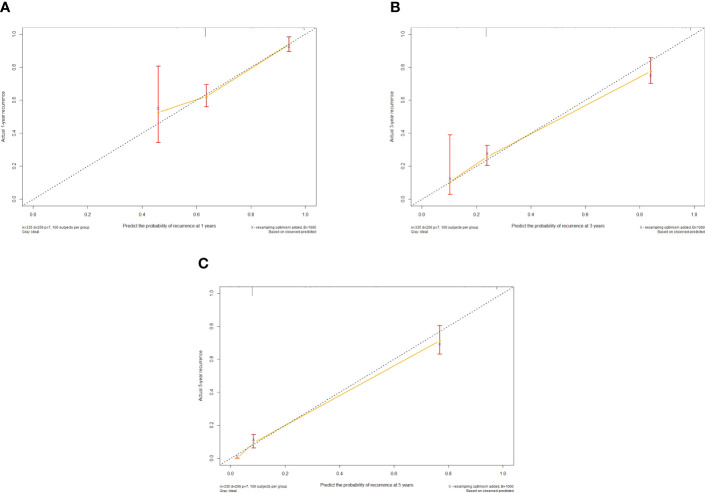
Calibration curve of the nomogram in the training cohort, with the x-axes actual recurrence estimated by the nomogram, the y-axes are observed recurrence calculated by the Kaplan-Meier method. **(A)** One-year RFS in the training cohort. **(B)** Three-year RFS in the training cohort. **(C)** Five-year RFS in the training cohort.

**Figure 4:**
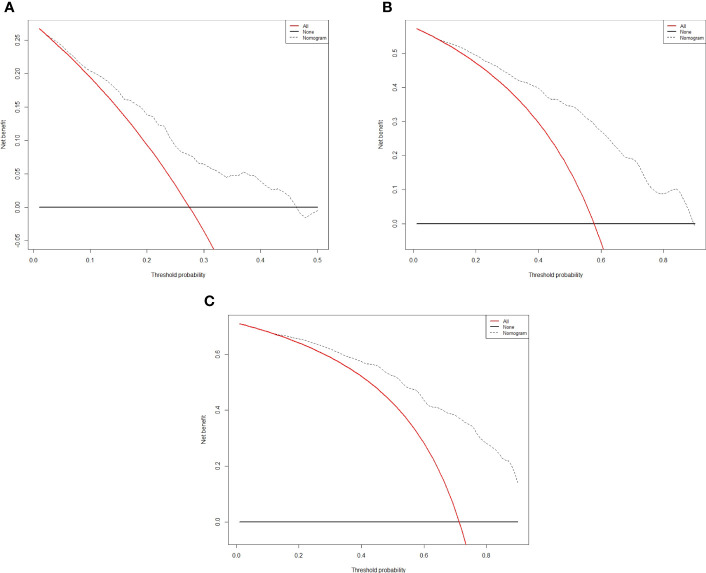
Decision curve analysis for recurrence in the training cohort. The x-axis indicate threshold probability, and the y-axis indicate the net benefit. Dashed lines: the net benefit of nomogram across a range of threshold probabilities. The solid red line: no patients relapse. The solid black line: all patients die or relapse. **(A)** Decision curve analysis for one-year RFS in the training cohort. **(B)** Decision curve analysis for three-year RFS in the training cohort. **(C)** Decision curve analysis for five- year RFS in the training cohort.

The independent predictors found by multi-factors Cox regression analysis were used to construct a nomogram (**Figure 1**). In the training cohort, the C-index was 0.717 (95%CI: 0.682-0.752), and the time-dependent ROC curve demonstrated that AUCs of 1-, 3-, and 5-year were 0.721, 0.825, and 0.845 (**Figure 2**). These results all indicated the real power of the nomogram for RFS. The calibration plot of the probability of 1-, 3-, and 5-year RFS reflected satisfactory accordance between the nomogram prediction and actual observation (**Figure 3**). Meanwhile, DCA curves were created to evaluate the clinical value of the nomogram (**Figure 4**), which showed encouraging net benefits in reasonable threshold probability with the nomogram.

**Figure 5:**
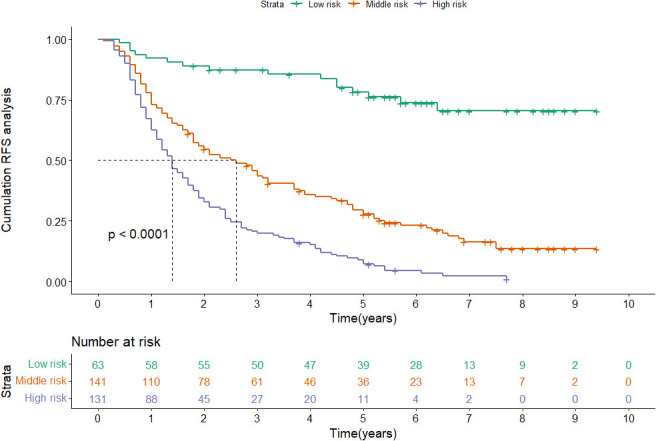
Kaplan-Meier plots of RFS for the low-risk group, medium-risk group and high-risk group in the training cohort.

According to the nomogram, patients were divided into low-risk, medium-risk, and high-risk groups (**Figure 5**). In the training cohort, there were apparent variances in RFS between the low-risk group (n=63), medium-risk (n=141), and high-risk group (n=131). There was distinct variance between the three groups (P< 0.001).

### 3.4 Validate the nomogram

To further confirm the reliability of the nomogram, we verified it internally. In the validation cohort, C-index was 0.752 (95%CI: 0.701-0.802), even higher than the training cohort, and the time-independent AUCs of 1-, 3-, and 5-year were 0.740, 0.868, and 0.837 (**Figure S1**). The calibration curves also matched well (**Figure S2**), and the DCA curves of 1-, 3-, and 5-year had good clinical practicability (**Figure S3**).

Patients in the validation cohort were categorized as low-risk (n=37), medium-risk (n=79), and high-risk (n=67) as well (**Figure S4**). Consistent with the training cohort, the risk of recurrence was obviously higher in the high-risk group than in the low-risk group and the medium-group (P< 0.001).

### 3.5 Comparison with other prognostic scores

We compared the predictive ability of the nomogram with five conventional prognostic scores. The results indicated that the nomogram exhibited better discriminatory ability, prominently higher than the other five scoring systems (**Table 3**).

**Table 3:** The AUROCs for predicting RFS of the nomogram and other prognostic biomarkers.

Prognostic biomarkers	AUROC for 1 year	AUROC for 3 years	AUROC for 5 years
Nomogram	0.721	0.825	0.845
PLR	0.612	0.645	0.671
NLR	0.713	0.742	0.769
MLR	0.698	0.727	0.748
GLR	0.619	0.632	0.653
ALBI	0.523	0.531	0.597

PLR, platelet to lymphocyte ratio; NLR, nrutrophil to lymphocyte ratio; MLR, Monocyte to lymphocyte ratio; GLR, gamma-glutamyl tranferase to lymphocyte ratio; ALBI, albumin-bilirubin.

## 4 Discussion

HCC remains a global health challenge, with incidence rates on the rise worldwide (1, 2, 10, 26). Because of the high recurrence rate, we need to devote more attention to the prognosis of HCC, in particular for AFP negative patients. Our study is the first to focus on dynamic changes in AFP to develop and validate a nomogram, which will hopefully predict the recurrence in N-P patients (negative at baseline and positive at relapse).

Since AFP was identified as a tumor marker for HCC, it has been used for diagnosis, prognosis, and surveillance of HCC (27). In addition to being a predictor, AFP has also been considered for immunotherapy and defining molecular classes of liver cancer (28–30). AFP levels have been proven to correlate with the presence of vascular infiltration and the degree of differentiation (30–33). Moreover, our team’s previous study showed that RFS was shorter in N-P patients who were characterized by multiple tumors, large tumor diameter, and elevated transaminases, representing high liver tumor invasiveness and poor liver function, leading to poor prognosis (15). Although N-P patients have specific clinicopathological characteristics and prognoses, most prediction models have been developed without considering the specificity of liver biological function in the group of patients and can not reflect an accurate forecast (16). Therefore, we aim to create a nomogram to predict recurrence and prognosis in N-P patients.

The nomogram contains seven factors to produce the probability of an individual specific clinical event, including gender, tumor size, tumor number, lymphocyte, DBIL, GGT, and prealbumin. The scores of the nomogram were obtained by drawing a vertical line at the location of the corresponding total score so that it intersected the three lines predicting the risk of recurrence, and the value shown at the intersection were predicted RFS at 1, 3, and 5 years. This nomogram performed with good predictive ability, as supported by the C-index of 0.717 and 0.752 for the training cohort and validation cohort, respectively. This was similarly demonstrated by the high AUCs of the time-dependent ROC curves for the training and validation cohorts, especially for the 3- and 5-year time points. The calibration curves displayed good agreements, suggesting predictive accuracy for our nomogram, and the DCA curves confirmed a high net clinical benefit rate. Patients were divided into three different risk groups according to the nomogram, and RFS was clearly different between all three groups (P<0.0001), which indicated that our nomogram had a better ability to distinguish N-P patients to determine the risk of relapse after ablation therapy.

There are two possible reasons why the male is a recognized risk factor for the recurrence of HCC: androgen receptor activation may promote HCC cell progression and invasion, while the endogenous metabolite estrogen inhibits tumor growth due to its anti-proliferative, pro-apoptotic, and anti-angiogenic activities (34–37). A key feature of cancer is tumor size. One study suggested that tumor size ≤3cm may be associated with hypofractionation and higher survival rates (38, 39). Because of metastasis and recurrence, patients with HCC have adverse prognoses. Multiple tumors are more prone to microvascular invasion (MVI) than a single tumor, which can lead to increased tumor recurrence after surgery (40, 41). DBIL can reflect liver function reserve, which is an important indicator of the prognosis of HCC (42). As we all know, inflammation can contribute to the poor prognosis of HCC by inducing lymphopenia (43, 44). However, we found that the poor prognosis of patients with increased lymphocytes may be due to the specific pathological features of HCC with dynamic changes in AFP, and further basic research is needed to confirm this hypothesis. GGT, a metabolite of glutathione, has been justified in animal HCC models that serum GGT levels increase with the process of hepatocarcinogenesis and promote tumor progression (45). Prealbumin can predict the risk of recurrence of HCC as a sensitive marker reflecting protein synthesis (46).

TACE is a palliative treatment with the recommendation for patients with intermediate-stage HCC by BCLC guidelines. For early-stage HCC, TACE can mark the tumor and achieve tumor downstaging, thereby declining the time and increasing the success rate of ablation (47). Ablation is the insertion of the electrodes into the tumor issue under the guidance of ultrasound, CT, and MRI, and generates frictional heat by applying high-frequency electrodes to the tissue to damage and destroy HCC cells, resulting in protein denaturation, loss of cell membrane integrity, mitochondrial dysfunction and inhibition of DNA replication (48–50). Both domestic and international studies and the previous study from our team showed that combination therapy by TACE and ablation improved overall and progression-free survival compared with TACE alone (15, 51–54). Thus, we used TACE sequential ablation treatment, whose efficacy can be further demonstrated in multiple centers to improve the quality of life and extend the overall survival of more patients with HCC.

The clinical indexes applied in this study cover a wide range, including demographics, liver function, tumor load, tumor markers, and inflammatory indexes, enabling a more comprehensive assessment of the patients. What’s more, the compositions of our nomogram were simple and easy to obtain, allowing the physician to evaluate the patient’s condition in a timely and effective manner. Furthermore, our data were dynamic, focusing not only on baseline information but also on changes in indicators after patients relapsed.

Despite the good predictive performance of the nomogram, our study has some limitations. First, bias was inevitable since this was a retrospective study, and future prospective studies are needed to further validate the results of the nomogram. But internal validation results reflected that our nomogram was accurate and reliable. Secondly, the number of N-P patients was limited. However, in general, some studies only researched the relationship between AFP and prognosis at baseline, and some only explored the prognostic value of AFP when it recurred. Finally, the population receiving locoregional therapy was the subject of our study, and the applicability of this nomogram to patients undergoing surgery and liver transplantation is uncertain. Therefore, more studies are needed to explore the application of our nomogram in patients receiving other treatments. Nevertheless, we used up to eight years of follow-up to create an accurate and reliable nomogram to better guide clinical practice for this group of HCC patients with dynamic changes in AFP.

## Conclusion

In summary, based on multivariable Cox regression analysis, we created an accurate and reliable nomogram to predict recurrence in patients with dynamic changes in AFP. The nomogram, including gender, tumor number, lymphocyte, DBIL, GGT, and prealbumin, demonstrated good predictive ability, which could be instrumental in guiding therapeutic decisions.

## Data availability statement

The raw data supporting the conclusions of this article will be made available by the authors, without undue reservation.

## Ethics statement

The study had been approved by the Ethics Committee of Beijing Youan Hospital, Capital Medical University. As a minimal-risk study in compliance with the Helsinki protocol, the requirement for informed patient consent was waived by the same ethics committee that approved the study (Beijing Youan Hospital, Capital Medical University), and all methods were performed in accordance with relevant guidelines and regulations. In accordance with state law and institutional requiements, written informed consent for participation was not required in this study.

## Author contributions

Conceived and designed the protocol: YZ. Collected data: QW and YS. Analyzed data: WQ and YX. Wrote the manuscript: YX and WQ. Critically revised the manuscript: QW and HZ. All authors contributed to the article and approved the submitted version.

## Supplementary material

Supplementary Figure 1 ROC curve of the nomogram in the validation cohort. **(A)** The AUC for 1-year RFS was 0.740 in the validation cohort. **(B)** The AUC for 3-year RFS was 0.868 in the validation cohort. **(C)** The AUC for 5-year was 0.837 in the validation cohort.

Supplementary Figure 2 Calibration curve of the nomogram in the validation cohort, with the x-axes actual recurrence estimated by the nomogram, the y-axes are observed recurrence calculated by the Kaplan-Meier method. **(A)** One-year RFS in the validation cohort. **(B)** Three-year RFS in the validation cohort. **(C)** Five-year RFS in the validation cohort.

Supplementary Figure 3 Decision curve analysis for recurrence in the validation cohort. The x-axis indicate threshold probability, and the y-axis indicate the net benefit. Dashed lines: the net benefit of nomogram across a range of threshold probabilities. The solid red line: no patients relapse. The solid black line: all patients die or relapse. **(A)** Decision curve analysis for one-year RFS in the validation cohort. **(B)** Decision curve analysis for three-year RFS in the validation cohort. **(C)** Decision curve analysis for five- year RFS in the validation cohort.

Supplementary Figure 4 Kaplan-Meier plots of RFS for the low-risk group, medium-risk group and high-risk group in the validation cohort.
