# Supplementary figures and images for: Development and validation of a nomogram to predict the recurrence of hepatocellular carcinoma patients with dynamic changes in AFP undergoing locoregional treatments
Source: Front Oncol. 2023 Aug 28;13:1206345. doi: 10.3389/fonc.2023.1206345 (PMC10494718; doi:10.3389/fonc.2023.1206345)

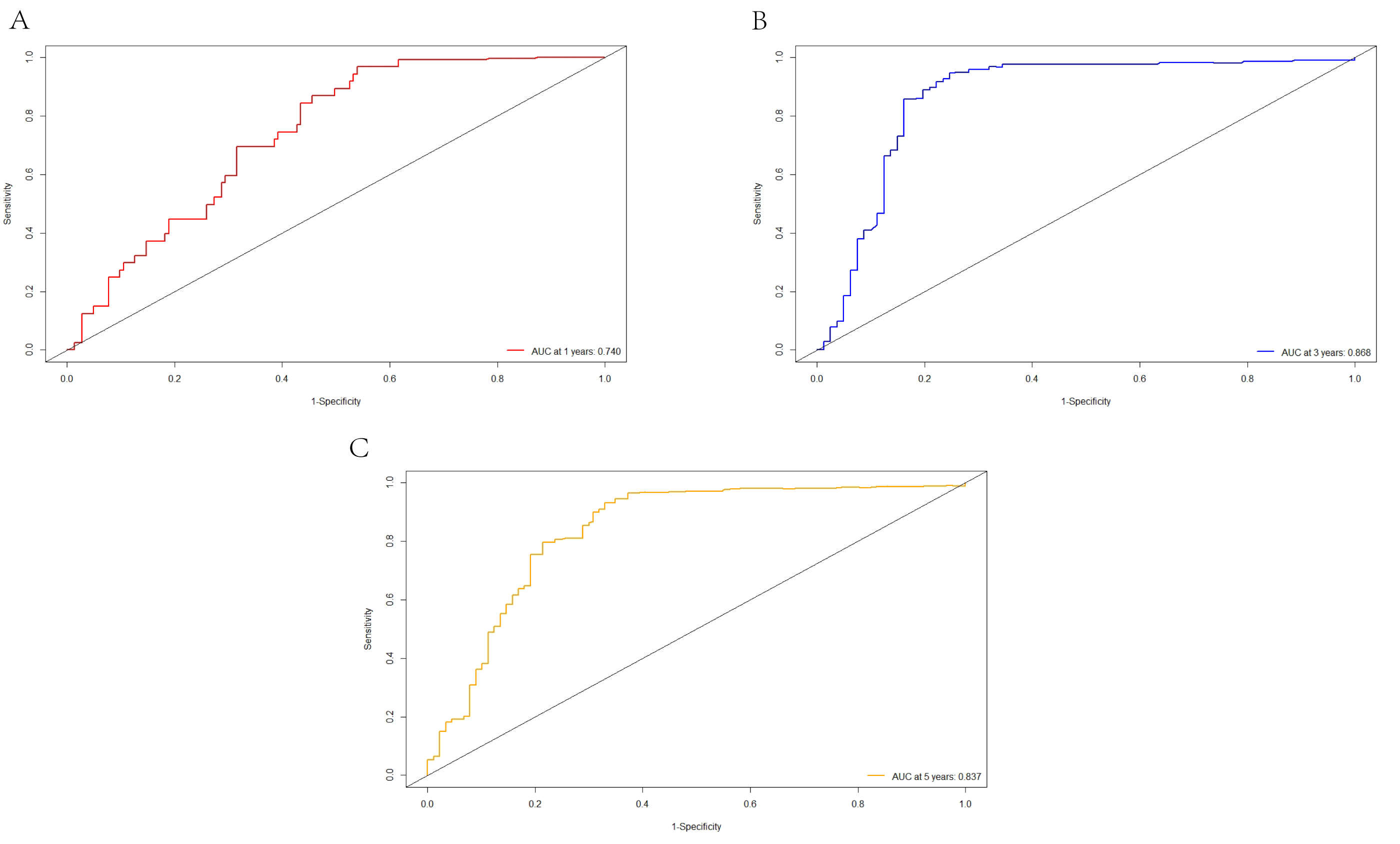

Supplement: Supplementary Figure 1 — ROC curve of the nomogram in the validation cohort. (A) The AUC for 1-year RFS was 0.740 in the validation cohort. (B) The AUC for 3-year RFS was 0.868 in the validation cohort. (C) The AUC for 5-year was 0.837 in the validation cohort. [file Image_1.tif]

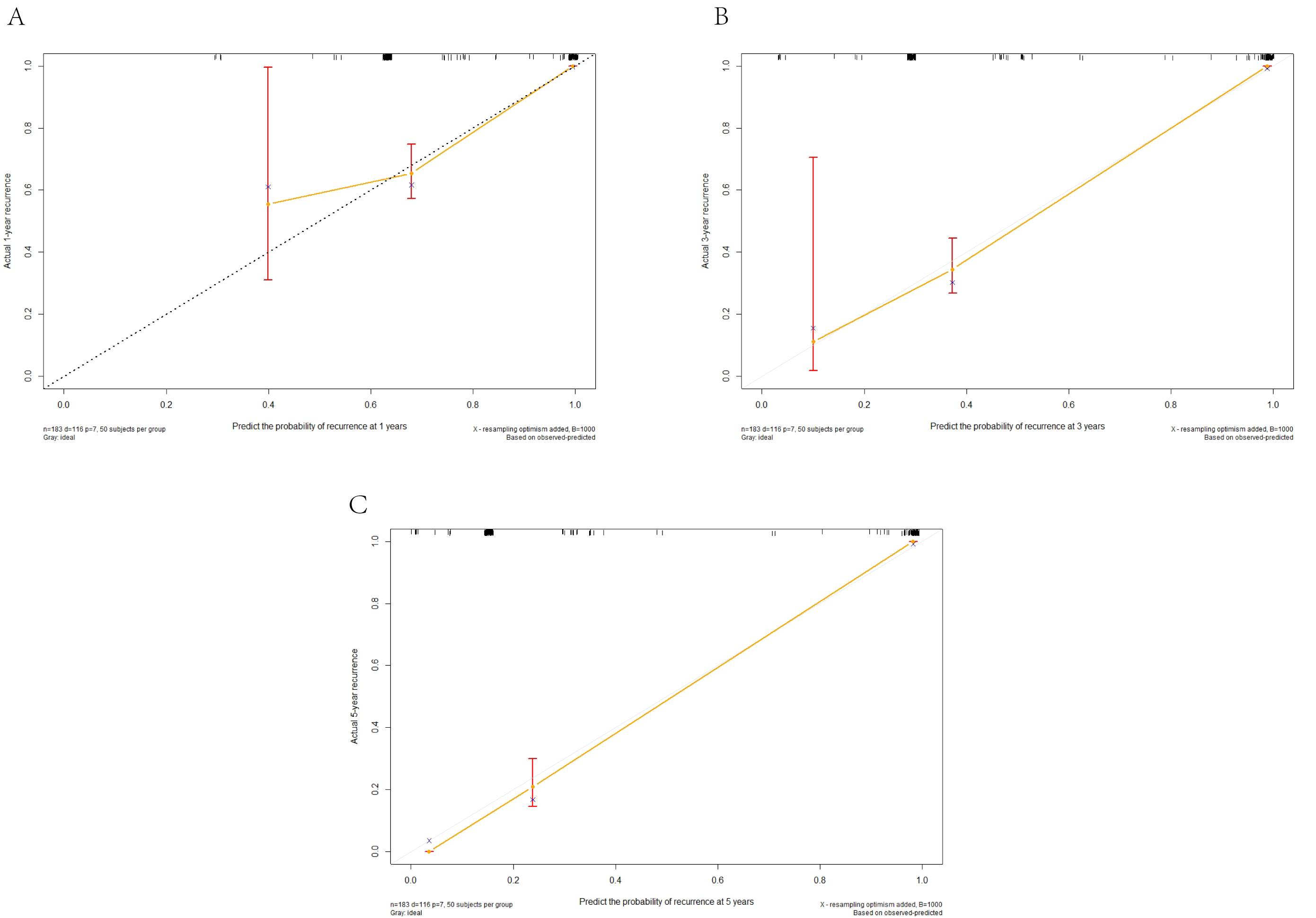

Supplement: Supplementary Figure 2 — Calibration curve of the nomogram in the validation cohort, with the x-axes actual recurrence estimated by the nomogram, the y-axes are observed recurrence calculated by the Kaplan-Meier method. (A) One-year RFS in the validation cohort. (B) Three-year RFS in the validation cohort. (C) Five-year RFS in the validation cohort. [file Image_2.tif]

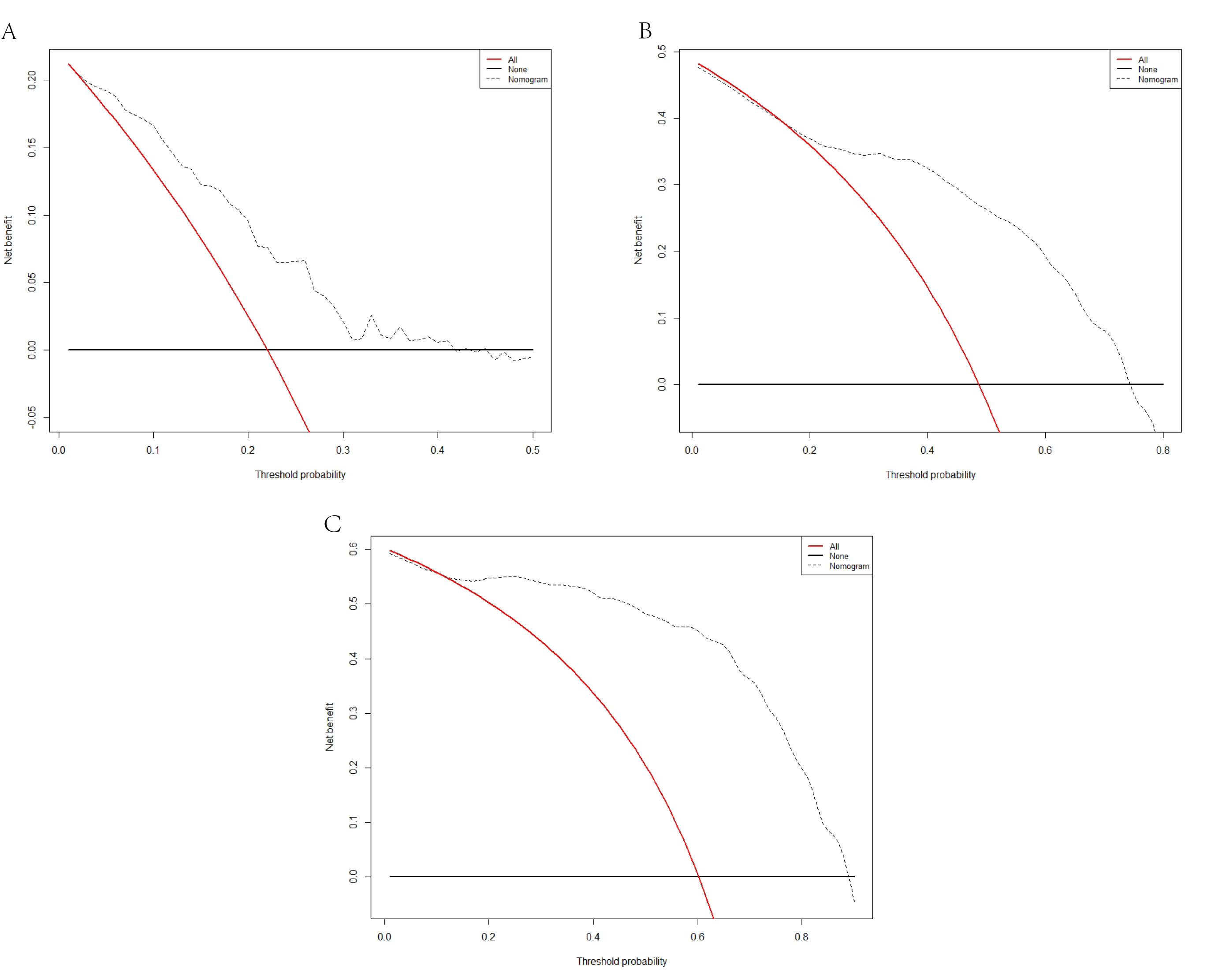

Supplement: Supplementary Figure 3 — Decision curve analysis for recurrence in the validation cohort. The x-axis indicate threshold probability, and the y-axis indicate the net benefit. Dashed lines: the net benefit of nomogram across a range of threshold probabilities. The solid red line: no patients relapse. The solid black line: all patients die or relapse. (A) Decision curve analysis for one-year RFS in the validation cohort. (B) Decision curve analysis for three-year RFS in the validation cohort. (C) Decision curve analysis for five- year RFS in the validation cohort. [file Image_3.tif]

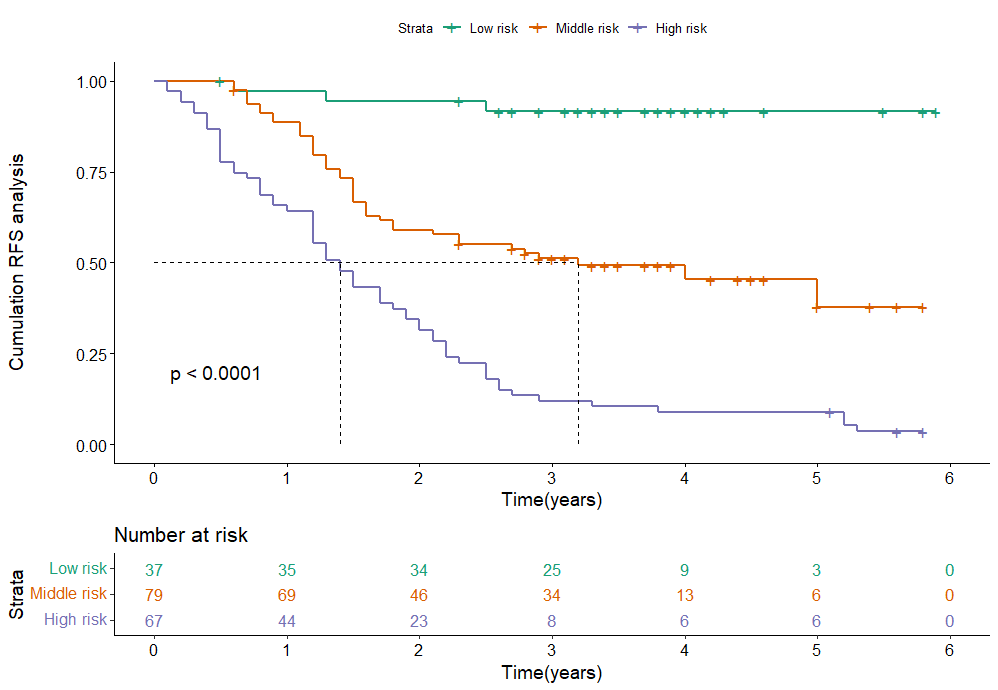

Supplement: Supplementary Figure 4 — Kaplan-Meier plots of RFS for the low-risk group, medium-risk group and high-risk group in the validation cohort. [file Image_4.tiff]
